# *XRCC3* Gene Polymorphism Is Associated with Survival in Japanese Lung Cancer Patients

**DOI:** 10.3390/ijms131216658

**Published:** 2012-12-05

**Authors:** Kayo Osawa, Chiaki Nakarai, Kazuya Uchino, Masahiro Yoshimura, Noriaki Tsubota, Juro Takahashi, Yoshiaki Kido

**Affiliations:** 1 Faculty of Health Sciences, Kobe University Graduate School of Health Sciences, Kobe 654-0142, Japan; E-Mails: evening-primrose@energy.ocn.ne.jp (C.N.); Jtaka-16-taka@S4.dion.ne.jp (J.T.); kido@med.kobe-u.ac.jp (Y.K.); 2 Department of General Thoracic Surgery, Hyogo Cancer Center, Akashi 673-0021, Japan; E-Mails: kazuya-uchino@hp.pref.hyogo.jp (K.U.); myoshi@hp.pref.hyogo.jp (M.Y.); 3 Department of Thoracic Oncology, Hyogo College of Medicine, Nishinomiya 663-8501, Japan; E-Mail: ntsubo@hm.h555.net; 4 Division of Diabetes and Endocrinology, Department of Internal Medicine, Kobe University Graduate School of Medicine, Kobe 650-0017, Japan

**Keywords:** gene polymorphisms, lung cancer, survival, DNA repair, XRCC3

## Abstract

We focused on *OGG1* Ser326Cys, *MUTYH* Gln324His, *APEX1* Asp148Glu, *XRCC1* Arg399Gln, and *XRCC3* Thr241Met and examined the relationship between the different genotypes and survival of Japanese lung cancer patients. A total of 99 Japanese lung cancer patients were recruited into our study. Clinical data were collected, and genotypes of the target genes were identified by polymerase chain reaction-restriction fragment length polymorphism (PCR-RFLP). Survival analysis to verify the impact of these gene polymorphisms on the clinical outcome of lung cancer showed that lung squamous cell carcinoma patients with the Thr/Met genotype at *XRCC3* had a significantly shorter survival time than those with the Thr/Thr genotype (13 months *versus* 48 months; log-rank test, *p* < 0.0001). Cox regression analysis showed that the carriers of *XRCC3* genotypes were at a significantly higher risk [adjusted hazard ratio (HR) = 9.35, 95% confidence interval (CI) = 2.52–34.68, *p* = 0.001; adjusted HR = 9.05, 95% CI = 1.89–44.39, *p* = 0.006]. Our results suggest that *XRCC3* Thr241Met may act as a favorable prognostic indicator for lung squamous cell carcinoma patients.

## 1. Introduction

Lung cancer is a major cause of cancer mortality worldwide. The 5-year survival rate for lung cancer, particularly non-small-cell lung cancer (NSCLC), remains at less than 20% [1,2]. Genetic factors are considered to influence the outcome of lung cancer. Among genetic factors, DNA repair capacity is an important factor. The reason appears to be that DNA repair pathways, including nucleotide excision repair (NER), base excision repair (BER), and double-strand break repair (DSBR), play an important role in maintaining genetic stability through different pathways [3,4]. It is also possible that DNA repair capacity can affect the survival of lung cancer patients.

Four key proteins in the BER pathway are 8-oxoguanine DNA glycosylase (OGG1), Mut Y homolog (MUTYH/MYH), apurinic/apyrimidinic endonuclease-1(APEX1/APE1), and X-ray repair cross-complementing group 1 (XRCC1). Among the various DNA repair pathways, BER is considered to play a key role in removing DNA damage resulting from exposure to various endogenous and exogenous carcinogens. OGG1 and MUTYH recognize and remove the misincorporated oxidized nucleotide 8-OHdG and the adenine paired with 8-OHdG, respectively, and also prevent the occurrence of these events. *OGG1* Ser326Cys is associated with the risk of lung cancer [5]. We have reported that *MUTYH* Gln324His was associated with increased risk of lung and colorectal cancers [6,7]. APEX1, the most stable product of oxidative DNA damage, exhibits 3′-phosphodiesterase activity that removes the abasic sites from cleaved DNA through OGG1 and MUTYH proteins [8]. Recently, we reported that genetic polymorphisms of *APEX1* in DNA repair pathways contributed to lung cancer susceptibility, which was dependent on smoking status [6,9]. XRCC1 encodes a protein that complexes with DNA ligase to repair DNA gaps resulting from BER, and a polymorphism at codon 399 Arg to Gln of *XRCC1* is associated with the risk of lung cancer.

In the DSBR pathway, the X-ray repair cross-complementing group 3 (XRCC3) is integral to DNA double-strand break recombination repair, and a polymorphism in codon 241 (Thr and Met) of *XRCC3* has been associated with the level of bulky DNA adducts in leukocytes of healthy subjects [10].

Recently, there has been increasing evidence that reduced DNA repair capacity resulting from genetic polymorphisms of various DNA repair genes is associated with improved survival with platinum-based chemotherapy [11,12].

To our knowledge, few previous studies have examined the effect of these polymorphisms on the association between outcome and lung cancer in Japanese patients without chemotherapy. To determine the significance of these polymorphisms, we focused on *OGG1* Ser326Cys (rs1052133), *MUTYH* Gln324His (rs3219489), *APEX1* Asp148Glu (rs1130409), *XRCC1* Arg399Gln (rs25487), and *XRCC3* Thr241Met (rs861539) and examined the relationship between the different genotypes and the survival of Japanese lung cancer patients.

## 2. Results

The distribution of characteristics and clinical features of 99 lung cancer patients are shown in Table 1. The average age [± standard deviation (SD)] of the patients was 66.3 ± 9.3 years, the average tumor size (± SD) was 36.4 ± 9.3 mm, and the average pack-years (±SD) was 34.7 ± 31.9 years. Histological analysis of samples from these patients showed that 65.4% had adenocarcinoma, 29.8% had squamous cell carcinoma, and 4.8% had other types of carcinoma. A total of 52 patients had died. The overall median survival time (MST) was 63 months. Region of metastasis mainly involved lung, bone, or mediastinum lymph node. As shown in Table 1, males; patients ≥65 years of age; whose histological subtype was squamous cell carcinoma; those who smoked; and those who had advanced cancer (stage III and IV), and who had T stage (T2-4), lymph node metastasis, or recurrence had significantly shorter MSTs (log-rank test, *p* < 0.05). The median survival was 52 months for males and 81 months for females (log-rank test, *p* = 0.001).

Multiple Cox regression analysis suggested that the risks of death from lung cancer were increased in patients with stages III and IV than in those with stages I and II (HR = 2.60, 95% CI = 1.42–4.75, *p* = 0.002), especially who had T stage (T2-4) and lymph node metastasis (HR = 3.68, 95% CI = 1.88–7.23, *p* < 0.0001 for T stage (T2&T3&T4); HR = 1.87, 95% CI = 1.05–3.33, *p* = 0.034 for lymph node metastasis; HR = 1.18, 95% CI = 0.37–3.79, *p* = 0.784 for distant metastasis). This analysis also showed that patients who were older (≥65 years of age), who smoked, whose histological subtype was squamous cell carcinoma, and who had recurrence had increased risks of death (HR = 2.75, 95% CI = 1.41–5.36, *p* = 0.003 for ≥65 years of age; HR = 1.93, 95% CI = 1.10–3.40, *p* = 0.023 for squamous cell carcinoma; HR = 3.02, 95% CI = 1.47–6.22, *p* = 0.003 for smoking status and HR = 6.15, 95% CI = 3.09–12.24, *p* < 0.0001 for recurrence). Females had significantly longer survival than males (HR = 0.32, 95% CI = 0.16–0.65, *p* = 0.001).

The associations between genotypes of 5 SNPs and survival of lung cancer patients are shown in Table 2. No associations were found between polymorphisms of these 5 genes and the overall survival of these patients. In the Cox regression mode, after adjusting for age, gender, tumor stage, metastasis, and recurrence, no associations were found between polymorphisms of these 5 genes and the overall risks of death of these patients.

**Figure 1:**
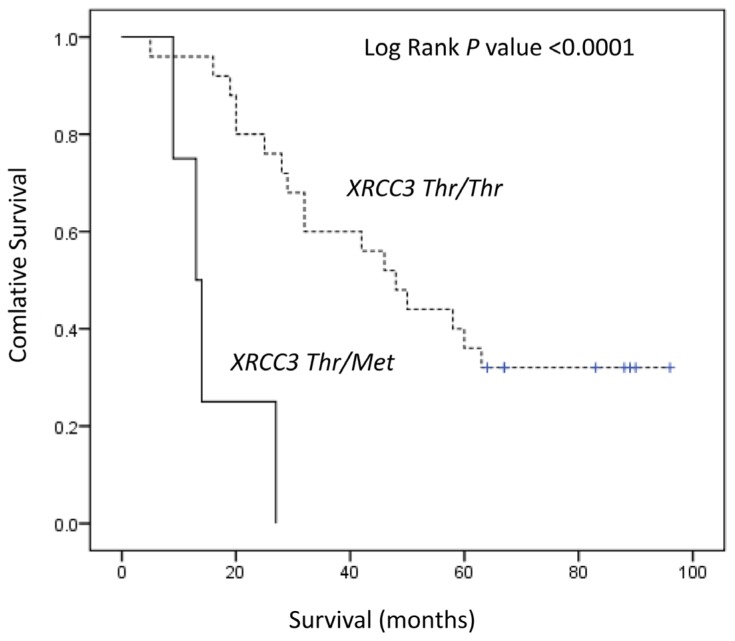
Kaplan–Meier survival curve of lung squamous cell carcinoma patients with the *XRCC3 Thr241Met* genotype.

Table 3 summarizes the genotype distribution for lung adenocarcinomas and squamous cell carcinomas. No associations were found between polymorphisms of these 5 genes and the risks of death for adenocarcinoma patients. For squamous cell carcinoma, patients with Thr/Met genotype at *XRCC3* showed a significantly shorter survival time than those with the Thr/Thr genotype (13 months *versus* 48 months; log-rank test, *p* < 0.0001) (Figure 1). Cox regression analysis showed that carriers of *XRCC3* had a significantly a higher risk (crude HR = 9.35, 95% CI = 2.52–34.68, *p* = 0.001; adjusted HR = 9.05, 95% CI = 1.89–44.39, *p* = 0.006).

## 3. Discussion

In this study, we assessed the *OGG1* Ser326Cys, *MUTYH* Gln324His, *APEX1* Asp148Glu, *XRCC1* Arg399Gln, and *XRCC3* Thr241Met gene polymorphisms that may influence DNA repair capacity and their association with the overall survival of lung cancer patients. The polymorphisms chosen for this study have also been shown to have functional significance and may be responsible for a low DNA repair capacity phenotype that is characteristic of cancer patients [13,14]. To our knowledge, this is the first report on these DNA repair gene polymorphisms in relation to survival without chemotherapy in Japanese lung cancer patients. In a previous study of Japanese patients, Takenaka *et al.* reported that the *ERCC1 C8092A* polymorphism may influence NSCLC prognosis regardless of ERCC1 protein expression and platinum sensitivity [15]. We explored the genotypes as well as pathological features of lung cancer patients in terms of their overall survival. In this study, *XRCC3* Thr241Met might be an independent prognostic factor in squamous cell carcinoma. The adjusted HR for *XRCC3* was 9.05 (*p* = 0.006), with the *XRCC3* group being significant. This *XRCC3* variant genotype was associated with significantly decreased survival in squamous cell carcinoma. In contrast, we observed that the patients carrying none of the adverse genotypes (*OGG1* Ser326Cys, *MUTYH* Gln324His, *APEX1* Asp148Glu, and *XRCC1* Arg399Gln) had much better survival than those carrying variant alleles. For BER genes, several studies reported that variant alleles of *XRCC1* 399 and *XRCC1* variant genotypes are significantly associated with poor survival [11,12,16–19]. In China, *ERCC1* and *XRCC1* were associated with the survival of non-smoker female lung adenocarcinoma patients [20]. Consistent with our study, Penas *et al.* showed that *XRCC 3* is strongly associated with the survival of NSCLC patients treated with cisplatin/gemcitabine [21]. They suggested that the reduced efficacy of the XRCC3 protein, a consequence of the polymorphic variant, may have resulted in an impaired ability to repair cisplatin DNA damage [22]. Another report showed that the *XRCC3* Met/Met genotype was significantly associated with increased risk of death among all patients, particularly males by univariate and multivariate analyses [23]. An explanation for these discordant results remains to be provided. However, there have been no previous reports on OGG1, MUTYH, and APEX1 with regard to survival in lung cancer. Thus, our report is the first on the detailed effects of DNA repair gene polymorphisms on the survival of Japanese lung cancer patients. In addition, numerous clinical features may play important roles in the survival of lung cancer patients. We found a significant association between survival and tumor histology. The disease stage at the time of diagnosis has a direct impact on the survival rate. Multivariate analysis showed that a higher stage (stages III and IV), male gender, older age, squamous cell carcinoma, smoking history, lymph node metastasis, and recurrence were independent prognostic factors associated with an increased risk of death. This higher tumor stage had a significant effect on survival, which is in accordance with other studies [18,24]. It is possible that individuals with these factors and with higher-stage disease already have too many genetic alterations during their tumor growth, which would reduce their survival. Our study has several limitations, especially the fact that the conclusion was based on several patients. Our data may be biased by the relatively small number of patients as a hospital-based case–control study. Therefore, further verification of these predictive biomarkers is required with a larger study population. The gene–environment interaction between smoking and these genotypes also needs to be clarified.

## 4. Experimental Section

### 4.1. Study Subjects

Study subjects included 99 lung cancer patients (65 with lung adenocarcinoma, 29 with lung squamous cell carcinoma, and 5 with other carcinomas) who were after surgical treatment but not receiving radiotherapy and chemotherapy and were included in previous studies that investigated the genetic polymorphisms of DNA repair proteins and metabolic enzymes [7,9,25]. These patients were recruited between April 2001 and July 2002 at the Hyogo Cancer Center. Informed consent was obtained from each patient. Detailed data on smoking were obtained by personal interviews. The study design was approved by the Ethics Review Committee on Genetic and Genomic Research, Kobe University Graduate School of Medicine. All samples were coded after the collection of blood and smoking frequency data. All patients were followed up for survival by November 2009 (the time of data analysis). Metastases were based on the status of pathological metastasis. The amount of smoke exposure was calculated as pack-years; the product of the number of years an individual smoked and the average number of cigarettes smoked per day (converted into a standard pack of 20 cigarettes).

### 4.2. Genotyping

Genomic DNA used for this study was isolated in previous studies [7,9,25]. The genotypes of *OGG1* Ser326Cys, *MUTYH* Gln324His, *APEX1* Asp148Glu, *XRCC1* Arg399Gln, and *XRCC3* Thr241Met were determined by polymerase chain reaction-restriction fragment length polymorphism (PCR-RFLP) analysis, as described previously [7,9].

### 4.3. Statistical Analysis

The effect of genetic polymorphisms on survival was also estimated using the Kaplan–Meier method and assessed using the log-rank test. The influence of clinical parameters on the outcomes of lung cancer patients was assessed by the log-rank test. The overall survival duration of lung cancer patients was calculated from the 1^st^ day of treatment until either death or the last follow-up. A multiple Cox regression model was used to obtain the adjusted hazard ratio (HR) and 95% confidence interval (95% CI) for potential prognostic factors in lung cancer patients. All *p* values were calculated from 2-tailed statistical tests. Statistical analysis was performed with the PASW software package (version 17.0 for Windows; SPSS Japan, Inc., Tokyo, Japan). A *p* value of <0.05 was considered significant for an association between a genotype and lung cancer.

## 5. Conclusions

We analyzed the association between polymorphisms of five DNA repair genes and the outcome of Japanese lung cancer patients. Our results suggest that the *XRCC3* Thr241Met gene polymorphism plays an important role in the overall survival of Japanese lung squamous cell carcinoma patients without chemotherapy. The *XRCC3* Thr241Met gene polymorphism may be a prognostic factor in lung squamous cell carcinoma patients.

## Figures and Tables

**Table 1:** Demographic and Clinical Characteristics of Lung Cancer Patients.

Variable	Patients *n*	Median Survival (Months)	Log-rank *p* value	Adjusted HR (95% CI)	*p* value
*Gender*					
Male	65	52	-	1.00	-
Female	34	81	0.001	0.32 (0.16–0.65)	0.001
*Age (years)*					
<65	36	78		1.00	
≥65	63	52	0.002	2.75 (1.41–5.36)	0.003
*Histological subtype*					
adenocarcinoma	65	67	-	1.00	-
squamous cell carcinoma	29	49	0.020	1.93 (1.10–3.40)	0.023
others	5	41	-	-	-
*Smoking status*					
Non-smokers (Pack-years = 0)	31	80	-	1.00	-
Smokers (Pack-years > 0)	67	53	0.002	3.02 (1.47–6.22)	0.003
No information	1	-	-	-	-
*Stage*					
I & II	74	69	-	1.00	-
III & IV	20	41	0.001	2.60 (1.42–4.75)	0.002
No information	5	15	-	-	-
*T stage*					
T1	39	82	-	1.00	-
T2&T3&T4	55	49	<0.0001	3.68 (1.88–7.23)	<0.0001
No information	5	15	-	-	-
*Lymph node metastasis*					
N0	66	68	-	1.00	-
N1&N2	28	49	0.030	1.87 (1.05–3.33)	0.034
No information	5	15	-	-	-
*Distant metastasis*					
M0	89	66	-	1.00	-
M1	5	50	0.783	1.18 (0.37–3.79)	0.784
No information	5	14	-	-	-
*Recurrence*					
No	50	83		1.00	
Yes	44	43	<0.0001	6.15 (3.09–12.24)	<0.0001
No information	5	4	-	-	-

**Table 2:** DNA Repair Gene Polymorphisms and Patient Survival.

Genotype	Patients *n*	MST (mon)	Log-rank *p* value	HR (95% CI)	Adjusted HR		

					*p* value	(95% CI) a	*p* value
*OGG1*							
Ser/Ser	25	58		1.00	-	1.00	-
Ser/Cys	50	70	0.910	0.88 (0.45–1.72)	0.712	0.96 (0.44–2.10)	0.927
Cys/Cys	24	63		0.99 (0.46–2.10)	0.972	0.90 (0.40–2.06)	0.808
Ser/Cys, Cys/Cys	74	66	0.784	0.92 (0.49–1.72)	0.786	0.94 (0.47–1.89)	0.854
*MUTYH*							
Gln/Gln	20	58		1.00	-	1.00	-
Gln/His	53	63	0.914	1.13 (0.55–2.33)	0.731	0.96 (0.42–2.17)	0.919
His/His	26	70		1.02 (0.45–2.32)	0.972	0.85 (0.33–2.18)	0.851
Gln/His, His/His	79	63	0.797	1.09 (0.55–2.18)	0.798	0.93 (0.42–2.03)	0.845
*APEX*							
Asp/Asp	40	58		1.00	-	1.00	-
Asp/Glu	48	62	0.649	0.88 (0.50–1.55)	0.652	1.02 (0.55–1.88)	0.963
Glu/Glu	11	71		0.61 (0.21–1.78)	0.37	0.51 (0.15–1.76)	0.289
Asp/Glu, Glu/Glu	59	64	0.505	0.83 (0.48–1.44)	0.508	0.91 (0.50–1.66)	0.766
*XRCC1*							
Arg/Arg	44	62	-	1.00	-	1.00	-
Arg/Gln	49	56	0.162	1.20 (0.69–2.08)	0.524	0.85 (0.46–1.55)	0.588
Gln/Gln	6	91		0.22 (0.03–1.62)	0.136	0.37 (0.05–2.87)	0.342
Arg/Gln, Gln/Gln	55	60	0.897	1.04 (0.60–1.79)	0.898	0.80 (0.44–1.46)	0.470
*XRCC3*							
Thr/Thr	88	66	-	1.00	-	1.00	
Thr/Met	11	28	0.202	1.67 (0.75–3.72)	0.209	1.94 (0.83–4.53)	0.128
Met/Met	0	-	-	-	-	-	-
Thr/Met, Met/Met	11	28	0.202	1.67 (0.75–3.72)	0.209	1.94 (0.83–4.53)	0.128

a HR adjusted for gender, age, smoking history, disease stage, metastasis, and reccurence.

**Table 3:** DNA Repair Gene Polymorphisms and Patient Survival in relation to Subtypes.

Genotype	Patients *n*	MST (mon)	Log-rank *p* value	HR (95% CI)	Adjusted HR		

					*p* value	(95% CI) a	*p* value
**Adenocarcinoma**							
*OGG1*							
Ser/Ser	16	62	-	1.00	-	1.00	-
Ser/Cys, Cys/Cys	49	68	0.784	0.89 (0.38–2.08)	0.785	0.80 (0.29–2.26)	0.803
*MUTYH*							
Gln/Gln	13	70		1.00		1.00	
Gln/His, His/His	52	66	0.65	1.25 (0.48–3.27)	0.653	1.54 (0.44–5.36)	0.498
*APEX*							
Asp/Asp	28	63	-	1.00	-	1.00	-
Asp/Glu, Glu/Glu	37	70	0.549	0.80 (0.39–1.67)	0.553	1.14 (0.48–2.73)	0.763
*XRCC1*							
Arg/Arg	26	71		1.00	-	1.00	-
Arg/Gln, Gln/Gln	39	64	0.522	1.28 (0.60–2.76)	0.526	0.87 (0.35–2.20)	0.775
*XRCC3*							
Thr/Thr	58	67	-	1.00	-	1.00	-
Thr/Met, Met/Met	7	64	0.995	1.00 (0.30–3.32)	0.995	1.32 (0.38–4.64)	0.661
**Squamous Cell Carcinoma**							
*OGG1*							
Ser/Ser	8	52	-	1.00	-	1.00	-
Ser/Cys, Cys/Cys	21	48	0.824	1.11 (0.43–2.88)	0.825	1.25 (0.44–3.55)	0.670
*MUTYH*							
Gln/Gln	5	64	-	1.00	-	1.00	-
Gln/His, His/His	24	45	0.377	1.72 (0.51–5.85)	0.385	4.73 (0.51–5.85)	0.153
*APEX*							
Asp/Asp	11	40		1.00	-	1.00	-
Asp/Glu, Glu/Glu	18	54	0.328	0.65 (0.27–1.56)	0.334	0.43 (0.14–1.32)	0.139
*XRCC1*							
Arg/Arg	16	50		1.00	-	1.00	-
Arg/Gln, Gln/Gln	13	46	0.991	1.00 (0.42–2.37)	0.991	1.17 (0.46–2.94)	0.743
*XRCC3*							
Thr/Thr	25	48	-	1.00	-	1.00	-
Thr/Met, Met/Met	4	13	<0.0001	9.35 (2.52–34.68)	0.001	9.05 (1.89–44.39)	0.006

a HR adjusted for gender, age, smoking history, disease stage, metastasis, and reccurence.

## Abbreviations

NER: nucleotide excision repair
BER: base excision repair
DSBR: double-strand break repair
OGG1: 8-oxoguanine DNA glycosylase
MUTYH/MYH: Mut Y homolog
APEX1/APE1: apurinic/apyrimidinic endonuclease-1
XRCC1: X-ray repair cross-complementing group 1
XRCC3: X-ray repair cross-complementing group 3
CI: confidence interval
NSCLC: non-small-cell lung cancer
SD: standard deviation
HR: hazard ratio
MST: median survival time
